# Role of metalloproteases in the CD95 signaling pathways

**DOI:** 10.3389/fimmu.2022.1074099

**Published:** 2022-12-05

**Authors:** Laurent Devel, Nicolas Guedeney, Sarah Bregant, Animesh Chowdhury, Mickael Jean, Patrick Legembre

**Affiliations:** ^1^ Université Paris-Saclay, CEA, INRAE, Département Médicaments et Technologies pour la Santé (DMTS), SIMoS, Gif-sur-Yvette, France; ^2^ Université de Rennes 1, Institut des Sciences Chimiques de Rennes - UMR CNRS 6226 Equipe COrInt, Rennes, France; ^3^ National Institute of Biomedical Genomics, Kalyani, West Bengal, India; ^4^ CRIBL UMR CNRS 7276 INSERM 1262, Université de Limoges, Rue Marcland, Limoges, France

**Keywords:** ADAM, CD95L, cancer, cleavage, inflammation, MMP

## Abstract

CD95L (also known as FasL or CD178) is a member of the tumor necrosis family (TNF) superfamily. Although this transmembrane ligand has been mainly considered as a potent apoptotic inducer in CD95 (Fas)-expressing cells, more recent studies pointed out its role in the implementation of non-apoptotic signals. Accordingly, this ligand has been associated with the aggravation of inflammation in different auto-immune disorders and in the metastatic occurrence in different cancers. Although it remains to decipher all key factors involved in the ambivalent role of this ligand, accumulating clues suggest that while the membrane bound CD95L triggers apoptosis, its soluble counterpart generated by metalloprotease-driven cleavage is responsible for its non-apoptotic functions. Nonetheless, the metalloproteases (MMPs and ADAMs) involved in the CD95L shedding, the cleavage sites and the different stoichiometries and functions of the soluble CD95L remain to be elucidated. To better understand how soluble CD95L triggers signaling pathways from apoptosis to inflammation or cell migration, we propose herein to summarize the different metalloproteases that have been described to be able to shed CD95L, their cleavage sites and the biological functions associated with the released ligands. Based on these new findings, the development of CD95/CD95L-targeting therapeutics is also discussed.

## Introduction

Different environmental factors (infection, pollution, UV …) involved in chronic inflammatory disorders and cancers affect the expression level and/or the interaction of different receptors and ligands, which in turn alter intracellular signaling pathways, subsequently leading to pathophysiological phenotypic changes. Death receptors (DR) are transmembrane receptors that can implement cell death signals *via* apoptosis, necroptosis, pyroptosis or ferroptosis. Ligands of the tumor necrosis factor (TNF) family and their receptors (TNF-R) are cytokines contributing to the induction of a caspase-dependent apoptotic death. Interestingly, these so-called “death receptors” can also trigger non-apoptotic signaling pathways involved in cell migration, differentiation, survival, and proliferation (1–5).

Six human death receptors (DRs) have been identified, TNF-R1 (6, 7), CD95 (Fas/APO-1/TNFRSF6) (8, 9), TRAIL-R1 (DR4) (10), TRAIL-R2 (DR5) (11, 12), DR3 (TRAMP) (13–16), and DR6 (also known as TNFRSF21 (17)). These death receptors are activated by TNF (18), CD95L (also known as FasL or CD178) (19), TRAIL (20), and TL1A, respectively (21), with the ligand for DR6 remaining to be confirmed even if amyloid precursor protein represents a solid option (22, 23). Apoptosis is finely regulated by these DRs, and mutations or expression deregulation of these receptors lead to various diseases (auto-immune, neurodegenerative, heart diseases or cancer) and development of chemoresistance (24).

## CD95 and CD95L

CD95 is a ubiquitously expressed transmembrane receptor, which belongs to the TNF-R family (8). Its natural ligand, CD95L is a transmembrane protein involved in the induction of a caspase-dependent apoptotic signal (8, 25, 26). The CD95/CD95L pair contributes to immune homeostasis and surveillance, and different mutations mainly localized within the CD95 death domain (DD), an intracellular region involved in the recruitment of the adaptor protein Fas-Associated protein with Death Domain (FADD), have been associated with breakdown of self-tolerance in autoimmune lymphoproliferative syndrome (ALPS) patients (27, 28) and Lpr^Cg^ mice (29, 30). CD95 mutations have also been detected in lymphoma pushing the authors to classify CD95 as a tumor suppressor gene (31, 32). Although DD-localized CD95 mutations foster tumor progression by rendering tumor cells resistant to the apoptotic response (33), new and accumulating evidence support that this receptor exerts more complex biological functions, and might promote oncogenesis and inflammation/auto-immunity independently of its ability to trigger cell death (3, 34–36).

For CD95L, rare mutations have been reported in human and are associated with lupus (37) or ALPS type Ib (38, 39) pathologies. The *gld* (for *generalized lymphoproliferative disease*) mice also display a lupus-like phenotype and harbor a mutation in CD95L with the replacement of its phenylalanine 273 by a leucine (F273L). This mutation reduces the efficiency of CD95/CD95L interaction (40).

Interestingly, CD95L might also interact with another TNFR member, DR5 (41). The authors show that, although the CD95L affinity for DR5 was weaker than that for CD95 ((K_D_ was 1.23x10^-12^ M for DR5–CD95L *versus* 6.01x10^-13^ M for DR5–TRAIL), CD95L can compete TRAIL for DR5 binding, suggesting that both ligands share a similar interaction region in DR5 (41). More importantly, the CD95L/DR5 interaction has been suggested to promote arthritis severity in a mouse model (*i.e.*, autoantibody-induced arthritis). Surprisingly, the K_D_ of CD95L for DR5 assessed in this study is far higher than that currently measured for CD95 (K_D_ comprised between and 7x10^-8^ and 2x10^-9^M (42–45), suggesting that CD95L would possess a stronger affinity for DR5 than for its own receptor. This conclusion remains to be strengthened with structural methods to definitively validate the CD95L/DR5 interaction.

**Figure 1:**
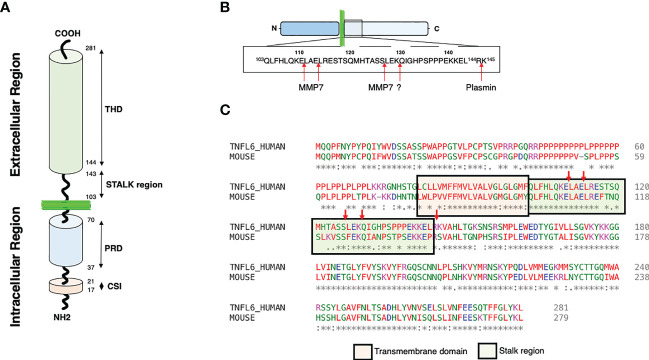
CD95L structures and cleavages sites. **(A)** Representation of CD95L domains. Proline rich domain: PRD; Casein kinase I substrate motif: CSI; TNF homology domain: THD. **(B)** Representation of the different cleavage sites described within the CD95L stalk region. **(C)** Alignment of human and mouse CD95L protein sequence using Clustal omega (1.2.4). The transmembrane and the stalk domains are represented.

At least, two main forms of CD95L exist. The transmembrane CD95L (m-CD95L) triggers cell death when it interacts with CD95-expressing cells, while metalloproteases can release soluble CD95L (s-CD95L) (46–48). Expressed by activated B and T-cells, m-CD95L contributes to the immune contraction (49) and its expression by myeloid cells participates in tissue inflammation by recruiting macrophage in damaged spinal cord (50). In this latter study, the role of m-CD95L and/or s-CD95L in the inflammatory process remains to be addressed. Contradicting studies exist on s-CD95L; while soluble CD95L can trigger apoptosis and promote lung damage in acute lung injury (ALI) (51, 52), it fails to induce cell death but rather stimulates inflammation in chronic autoimmune disorders such as systemic lupus erythematosus (SLE) (34, 48) and metastasis occurrence in cancers (53–57). Such a discrepancy might be ascribed to the stoichiometry of s-CD95L (43, 58), which seems to rely on the presence or absence of juxtamembrane N-terminal end (51, 59). In this respect, metalloproteases involved in the m-CD95L shedding as well as their preferential cleavage sites within the stalk region will directly impact the N-terminal length of s-CD95L (**Figures 1A** **–C**) end and thereby, its biological function as discussed below. It has been reported that m-CD95L can be shed close to its transmembrane domain releasing a s-CD95L encompassing a stalk region both in mouse (43, 60) and human (43, 61). This stalk region promotes the aggregation and the cytotoxic activity of s-CD95L. These observations point out that the presence or absence of certain metalloproteases involved in the CD95L shedding, might be responsible for the release of different ligands that either trigger cell death or aggravate inflammation or oncogenesis.

### Cloning

CD95L/FasL, cloned in 1993 (19), is a type II transmembrane protein that belongs to the tumor necrosis factor (TNF) family. Northern hybridization revealed that the ligand is mainly expressed in activated splenocytes and thymocytes, consistent with its involvement in T cell-mediated cytotoxicity and immune homeostasis. This ligand is also detected in several nonlymphoid tissues, such as testis (19). In 1989, a monoclonal antibody (mAb) APO-1 isolated by Peter Krammer’s group killed many tumor cells (25). This antibody recognizes CD95, a transmembrane receptor cloned in 1991 by the Nagata’s team (8, 9).

### CD95L and CD95 structures

As aforementioned, CD95L is a type II transmembrane protein that encompasses a long cytoplasmic intracellular domain, a transmembrane (TM) domain, a stalk region and a TNF homology domain (THD) (**Figure 1A**). The THD adopts a ‘jelly-roll’ topology that participates in the ligand homotrimerization and its interaction with CD95 (62). CD95L can be cleaved within its stalk region (amino acid residues 103 to 143) by different proteases (**Figure 1B**). Of note, only 3 cleavage sites over 5 are conserved between human and mouse (**Figure 1C**) suggesting that either different proteases or different sites are involved between these two species or that the main cleavage sites correspond to the three conserved sequences. The intracellular N-terminal region of CD95L is long and contains different domains including a casein kinase I (CKI) substrate motif (SSASS in human) and a proline-rich domain (PRD) (63)(**Figure 1A**). CD95L PRD interacts with proteins containing SH3 and/or WW domains (*i.e.*, SH3 domain of Src kinase p59^Fyn^) and these interactions seem to regulate the expression level and stability of CD95L (64, 65). In addition, PRD contributes to the CD95L-mediated reverse signaling (66, 67). Like TNF (68), the CKI domain of CD95L might also participate in the reverse signaling. In addition, the intracellular region of CD95L can be cleaved by signal peptide peptidase-like 2a (SPPL2a) releasing an intracellular peptide, trafficking to the nucleus to inhibit transcription (69). The biological role of SPPL2a cleavage and its cleavage site remain to be elucidated.

CD95 contains three extracellular cysteine-rich domains (CRDs) (70). While CRD1 is responsible for pre-association of the receptor at the plasma membrane and has been named the pre-ligand binding assembly domain (PLAD) (71–73), both CRD2 and CRD3 regions contribute to ligand binding (74). Although CD95 does not possess any enzymatic activity, its cytosolic region encompasses a death domain (DD) (75) involved in the apoptotic signal, and a juxtamembrane domain interacting with ezrin (76) and phospholipase Cγ1 (48, 77, 78) to promote neurite growth or cell migration, respectively. Through protein-protein interactions (PPIs), the 80-amino acid containing DD recruits the Fas-Associated protein with Death Domain (FADD), which in turn binds and aggregates the pro-caspase-8 (79). This complex, designated death inducing signaling complex (DISC), initiates apoptosis (79). The juxtamembrane region interacts with different partners to trigger the motility-inducing signaling complex (MISC) formation implementing a Ca^2+^ response, and the subsequent induction of non-apoptotic signaling pathways (2, 76, 80, 81).

## Extracellular matrix and metalloproteases

Extracellular matrix (ECM) is composed of a large number of structural and functional components that includes enzymes, collagens and proteoglycans, which are secreted and self-assembled into the immediate cellular environment (82). Other non-proteoglycan matrix components include hyaluronic acid, fibronectin, elastin, and laminin. This ECM provides structural support to cells and an integral signaling network through the action of different cytokines and growth factors interacting with the matrix components (83–86). For instance, binding of the s-CD95L to ECM, and more specifically to fibronectin, transforms the non-apoptotic ligand into a potent death inducer (87) suggesting that immobilization and/or aggregation of the s-CD95L homotrimer can foster the induction of the apoptotic response. In agreement with this observation, although a soluble and homotrimeric CD95L fails to trigger apoptosis, its hexameric counterpart (58) can do it. We also observed that the more CD95L is aggregated, the more its ability to induce apoptosis is increased (88).

Most of the ECM protein components are processed by matrix metalloproteinases (MMPs). In human, this family of zinc-dependent endopeptidases englobes 23 members sharing structural domains (89, 90). These proteases are mainly secreted within the pericellular and extracellular space (61) but can also be anchored to the cell surface (91) or adopt an intracellular localization, that has been correlated in certain cases to non-proteolytic functions (90, 92). Except during specific stages of development involving tissue remodeling (*e.g.*, embryogenesis) and wound healing processes, there is no constitutive expression of MMPs at homeostasis. Once secreted, these enzymes coexist within the extracellular space under latent forms (zymogens) and active forms, whose proteolytic activity is finely tuned by endogenous inhibitors such as tissue inhibitors of metalloproteases (TIMPs) or alpha-macroglobulin.

Recent N-terminomics and proteomics techniques have been used to profile hundreds of cleavage sites in proteomes associated with MMP activity, which reveal that more than two-third of MMP substrates are non-ECM proteins. Accordingly, far beyond their capacity to collectively cleave the ECM substrates, MMPs can process chemokines, cytokines, cell-surface receptors, growth factors, and nuclear proteins. Thus, MMPs are involved in inflammatory response, angiogenesis, cell-to-cell communication and cell proliferation, and the deregulation of their activity contributes to the progression of many diseases including cancer, chronic inflammatory disorders, vascular and central nervous system diseases (90).

**Figure 2:**
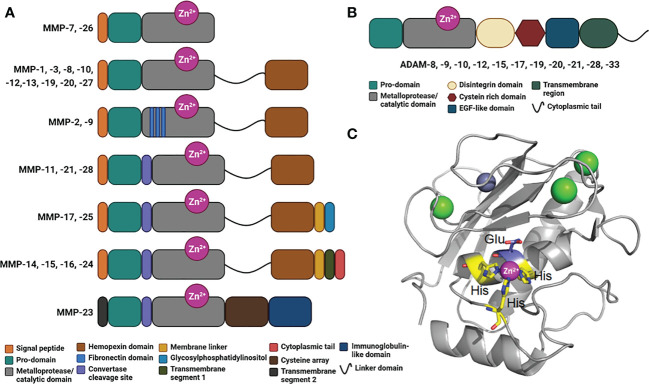
Domains in human MMPs and ADAMs. **(A)** Schematic representation of the domains in human MMPs consisting in a signal peptide, a prodomain, a metalloprotease/catalytic domain, a linker domain, a hemopexin domain, fibronectin inserts, a convertase cleavage site, a membrane linker, a glycosylphosphatidylinositol, a transmembrane segment 1, a cytoplasmic tail, a transmembrane segment 2, a cysteine array and immunoglobulin-like domain. **(B)** Schematic representation of ADAMs organized in modules consisting in a prodomain, a metalloprotease/catalytic domain, a disintegrin domain, a cysteine rich domain, an EGF-like domain, a transmembrane region and a cytoplasmic tail. **(C)** Crystal structure of a typical Metalloprotease/catalytic domain in cartoon representation (hMMP-12, PDB code: 4GQL), with catalytic zinc ion as magenta ball, His residues chelating the catalytic zinc ion in yellow stick, catalytic glutamic acid residue in blue stick, and structural zinc and calcium ion as grey and green balls, respectively.

MMPs are classified according to their linear sequence similarity, domain organization and substrate specificity (90). All the MMPs share a minimal N-terminal region, consisting in a signal peptide, a pro-domain and a metalloprotease/catalytic domain (90) (**Figure 2A**). Except for MMP-7, -26 and -23, all MMPs encompass a hemopexin-like C-terminal region, which is important in determining substrate specificity and interaction with tissue inhibitors of metalloproteinases (TIMPs). This C-terminal domain plays also an important role in cell migratory function of certain MMPs. Gelatinase-A (MMP-2) and gelatinase-B (MMP-9) contain fibronectin type-II inserts within their catalytic domain. These inserts confer the ability to bind and cleave gelatin and collagen.

Membrane-type MMPs (MT-MMPs) are embedded in the plasma membrane of the cells *via* a transmembrane domain or a glycosylphosphatidylinositol (GPI)-anchor (**Figure 2A**). This family includes the transmembrane proteins MMP-14, MMP-15, MMP-16, and MMP-24, and the GPI-anchored proteins MMP-17 and -25 (93). Some MMPs harbor a furin-like convertase cleavage site (**Figure 2A**), which is intracellularly cleaved to activate the protease and promote its distribution at the cell surface. MMP-23 is a unique MMP that contains a cysteine array and immunoglobulin-like domain, whose exact role remains elusive.

Within the extracellular space, a disintegrins and metalloproteinases (ADAMs) family can also exert a proteolytic activity (94–96). The main substrates for ADAMs are type I and II transmembrane proteins, which make them as shedding proteases. However, these proteases are also capable of processing cytokines and growth factors (95). Interestingly, in the case of transmembrane proteins, the cleavage consistently occurs between 10 and 15 amino acids from the plasma membrane. Like MMPs, ADAMs possess several domains, including a pro-domain, a metalloprotease/catalytic domain, a disintegrin domain, a cysteine rich domain, an EGF-like domain, a transmembrane domain and a C-terminal cytoplasmic tail (**Figure 2B**). All ADAMs contain a disintegrin domain, which can bind to integrins from adjacent cells, with potential consequences in cell adhesion and migration. These metalloproteases are implicated in different diseases including cancer (95), systemic inflammation (96), cardiovascular diseases and atherosclerosis (97, 98). A critical role in kidney pathologies (99) and in immunity (100) has also been documented.

Both MMPs and ADAMs belong to the superfamily of metzincin proteases. These metzincins share a conserved HEXXHXXGXXH motif within their metalloprotease/catalytic domain, where the three histidine residues bind to the catalytic zinc ion and the glutamate, as a general acid base, and activates a water molecule required for the peptide bond hydrolysis (**Figure 2C**).

## MMPs, ADAMs and CD95L regulation

CD95L can be cleaved by several metalloproteases, including MMPs and ADAMs, to release different soluble CD95Ls (s-CD95Ls), which have been reported to induce cell proliferation, migration, survival (36) but also cell death (51, 59). Rendering more complex to predict the biological outcome of s-CD95L, this ligand can also interact with other TNFR members, including as aforementioned, DR5 (41) or the soluble receptor DcR3 (44). Despite the complexity of the signaling pathways induced by the different forms of s-CD95L and their implication in the progression of different pathologies including chronic inflammatory disorders and cancers only a limited structural knowledge exists on these s-CD95Ls.

### Metalloproteases and CD95L

Thirty years after CD95L cloning, it remains difficult to address what are the MMP/ADAMs responsible for the cytokine shedding, where the protease cleaves m-CD95L and whether the released soluble factor triggers non-apoptotic (34, 41, 55, 57, 78, 80, 101) or apoptotic outcome (51, 59, 102).

Some ADAM members have been described to contribute to the generation of s-CD95L. Indeed, both ADAM10 (69, 103) and ADAM17 (104) can cleave m-CD95L to release s-CD95L. ADAM10 can also shed the transmembrane TNFα (105). As aforementioned, a second step occurs following ADAM10-mediated cleavage, with the SPPL2a-mediated cleavage of the CD95L intracellular region to release a cytosolic domain modulating gene expression (69). MMP7 also cleaves the transmembrane CD95L but the biological role of the released ligand remains difficult to apprehend. While from prostate epithelial cells, MMP7 can release a soluble and cytotoxic CD95L, which is involved in the involution of the organ in rat (106), the same metalloprotease in human sheds membrane-bound CD95L from tumor cells to protect them from doxorubicin or oxaliplatin-induced cell death in human (107, 108). S-CD95L is increased in sera of human idiopathic pulmonary fibrosis (IPF) and bleomycin-induced lung fibrosis in mice and this ligand prevents the elimination of fibrotic-lung myofibroblasts by CD95L-expressing T cells (109). Accordingly, MMP-7 knock-out mice exhibit resistance to the bleomycin-induced lung fibrosis, probably because these animals fail to cleave CD95L and generate the anti-apoptotic soluble ligand (109). Of note, MMP7 also cleaves the receptor of CD95L, CD95 and by doing so, promotes its ability to implement non-apoptotic signaling pathways in cancer cells (45, 110).

Regarding the cleavage positions within the CD95L stalk region, *in vitro* analyses revealed that MMP-7 is likely to cleave before the two leucine residues in the amino acid residues ^110^ELAELR^115^ conserved between human and mouse sequences (**Figures 2B, C** **)** (111). This sequence is at proximity of the plasma membrane bilayer suggesting that the released ligand might exert an apoptotic function because it conserves a full-length stalk region. As above mentioned, the stalk region of CD95L seems to exert a pivotal role in the apoptotic property of the soluble ligand (59). For instance, conservation of the stalk region (**Figures 1A, C** **)** in the soluble CD95L dosed in acute respiratory distress syndrome (ARDS) engenders a cytotoxic ligand killing the alveolar epithelial cells by apoptosis (51). Mutations of the ^110^ELAELR^115^ sequence do not completely abrogate the release of s-CD95L, because MMP7 might process m-CD95L at an additional position between ^126^SL^127^ (111), which, in this case, generate a non-apoptotic cytokine regarding the loss of the stalk region. Tschopp’s team also highlighted a cleavage of the transmembrane CD95L between amino acid residues ^126^SL^127^ (47), while Nagata’s team observed a processing between ^129^KQ^130^ (46, 112). The protease(s) involved in these shedding was/were not identified and although the cleavage sites diverge, both groups came with the conclusion that the metalloprotease-cleaved CD95Ls do not trigger apoptosis.

In rheumatoid arthritis (RA), MMP3 has also been suggested to cleave m-CD95L and accumulate s-CD95L in the synovial fluid of these patients (113). The role of s-CD95L in RA remains to be elucidated.

In neuronal and glial cells, preclinical studies showed that MMP9 contributes to the motor neuron cell death in amyotrophic lateral sclerosis (ALS) patients by regulating TNF-α and CD95L expression (114). Selective inhibition of MMP-9 activity has also been shown to increase in the m-CD95L/s-CD95L ratio on neonatal monocytes (115). Macrophages exposed to bacteria (*i.e.*, Escherichia coli infection) undergo an increase in CD95L expression (115) and the up-regulation of MMP-9 in these cells protects them from an autocrine and/or paracrine precocious phagocytosis-induced cell death by shedding the transmembrane CD95L.

Plasmin, a serine protease, can also cleave CD95L between amino acid residues Arg^144^ and Lys^145^ (**Figure 2B**) and although the released CD95L is devoid of its stalk region, it can still trigger cell death in endothelial cells (102). In conclusion, not only the identification of the amino acid sequence, but also the structure and stoichiometry of the soluble CD95Ls present in the different chronic inflammatory disorders and cancers must be realized to apprehend the biological role of each CD95 ligand.

### MMPs and cancer

Many studies have reported the expression of MMPs in human cancers. However, what was originally thought about their detrimental roles has been challenged these two last decades. Indeed, an overexpression of certain MMPs does not necessarily imply the promotion of tumor or metastasis. In this respect, at least 10 MMPs have been reported to have protective roles in cancer (116). Among the “oncogenic” MMPs, MMP-2 and MMP-9 have been implicated as the most important prognostic factor in cancer microenvironment (117, 118). MMP-2 is correlated with the development of different types of cancers and associated with poor prognosis (119, 120). MMP9 contributes to the ECM remodeling and the release of membrane-bound proteins and thereby, might favor cell invasion and poor prognosis (121, 122). Other MMPs such as MMP3, MMP-7, MMP-11, and MMP-13 also participate in cancer development (123–128). With MMPs, ADAM10 is up-regulated in gastric cancer lesions compared with adjacent non-cancerous tissues (129). It remains to evaluate whether these metalloproteases could affect oncogenesis by reducing the quantity of membrane-bound CD95L or increasing the concentration of soluble CD95L. Numerous small-molecule MMP inhibitors (MMPi) have been developed but systematically failed in late-stage clinical studies (91, 130). Beside their poor pharmacokinetics and low oral availability/inability, this major failure has been mainly attributed to their lack of specificity within the MMP family and towards other metalloenzymes. Benefiting from a better understanding of MMP biology that emphasizes the necessity to selectively target one single MMP in a given pathological context, a new generation of selective MMPi has emerged recently (131). To achieve a better selectivity, several strategies have been deployed. Regarding the small-molecule inhibitors they mainly consist in either replacing the hydroxamic acid group found in most of broad spectrum MMPis by a weaker Zn^2+^ chelating moiety (132, 133) or targeting exclusively the S1’ pocket which significantly differ between the MMPs (131, 132). Alternatively, the development of surrogates of MMPs endogenous inhibitors such as TIMP analogs or targeting MMP gene expression using mRNAs have also been explored. Despite these improvements, finding the right balance between activity, selectivity and ADMET parameters still remain challenging and the timing of MMPi application is critical to achieve the desired therapeutic effect, as the “window of opportunity” is often in premetastatic disease (91, 130, 134).

### CD95L, metalloproteases and cancer

Accumulating evidence highlight the pro-oncogenic role of CD95 and CD95L pair. Although the elimination of CD95 expression in some colorectal tumors was reported to predict metastatic tumor recurrence (135), most of the analyses indicate that CD95 expression is maintained in these tumors and contributes to activate pro-oncogenic signaling pathways (136). On the other side, the expression of membrane CD95L and CD95 expression is gradually increased during progression from (early) adenoma to colorectal carcinoma (56, 137). Overexpression of CD95 in apoptosis-resistant 3LL cells makes them apoptosis-sensitive *in vitro* (138) but, transplantation of these cells into mice, reveals a tumor growth advantage as compared to control cells. This underscores the importance of investigating a mechanism within an environment that resembles the clinical situation as much as possible. The seminal experiments establishing the oncogenic role of CD95 came from the elimination of the receptor in two mouse models of cancers (*i.e.*, ovarian and liver cancers), which was associated with the significant reduction of cancer occurrence and growth (35). More recently, we observed that the expression of CD95 is maintained in triple negative breast cancer (TNBC) cells to regulate the NF-κB signaling pathway (139). Accordingly, CD95 loss in TNBC cells stimulates an inflammatory signal, which contributes *in vivo* to the anti-tumor activity of natural killer (NK) cells (140). Therefore, although soluble CD95L is an attractive target to develop drugs and prevent metastasis dissemination of TNBC cells (57), it might be more appropriate to develop therapeutics targeting CD95.

Accumulating evidence support that s-CD95L promotes tumor development and metastasis but the MMPs or ADAMs involved in this process remain to be elucidated. The identification of i) the MMPs/ADAMs and ii) their cleavage sites in CD95L will help us to identify how many s-CD95Ls exist *in vivo*, and anticipate their stoichiometry to better predict their biological effects on the immune response and the tumor progression.

## Targeting CD95/CD95L in clinic, what next?

**Figure 3:**
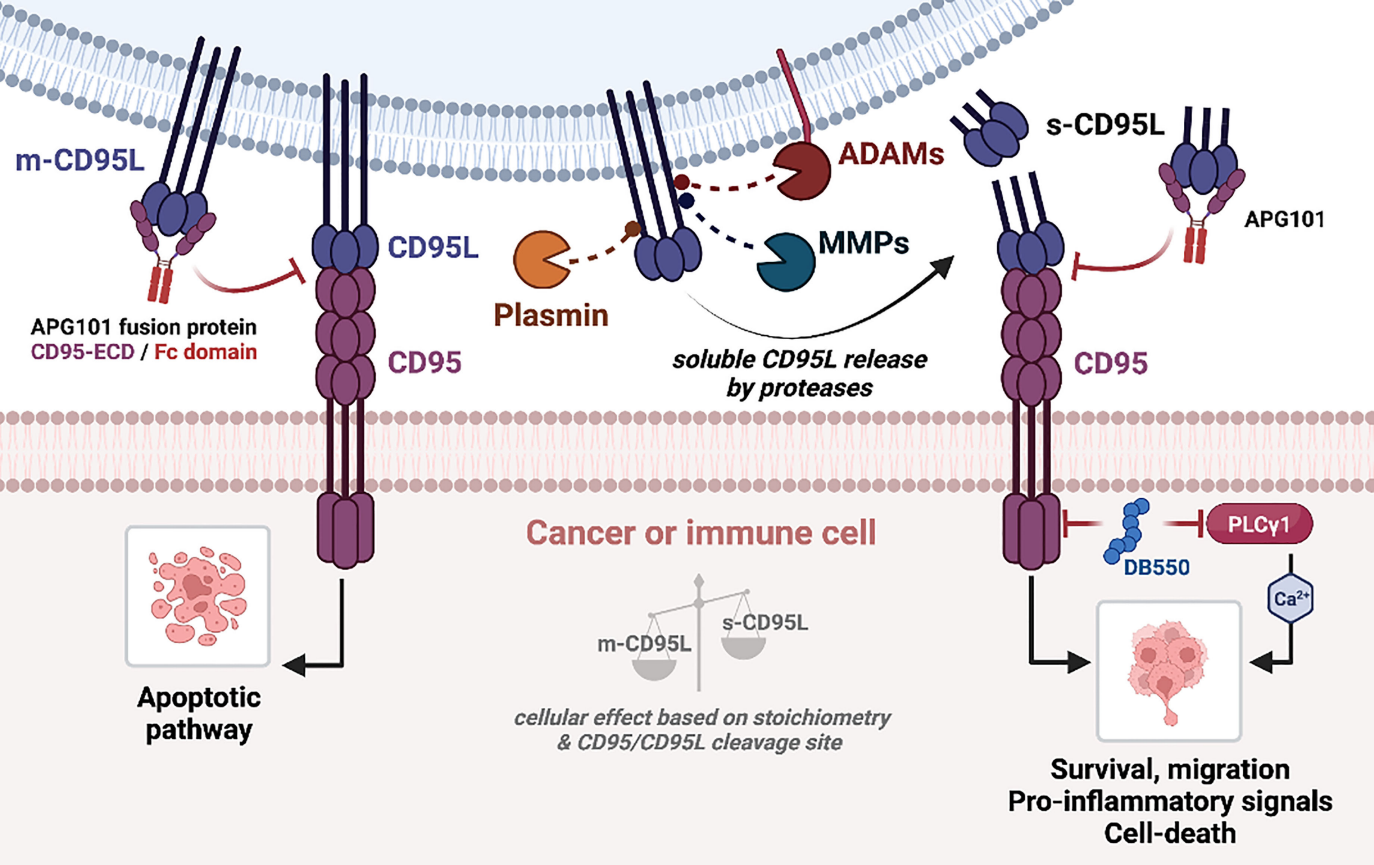
CD95/CD95L-mediated signaling pathways. (Left) Binding of m-CD95L to CD95 induces an apoptotic signaling pathway. (Right) m-CD95L processing by proteases (ADAMs, MMPs, plasmin) leads to the release of different s-CD95L in the extracellular environment. Depending on the ratio m-CD95L/s-CD95L, and the shedding sequence, several signaling pathways can be triggered: cell survival, migration (promotes the development of metastases), chemoattraction and pro-inflammatory signal, or cell death. Blocking of CD95L binding to CD95 by APG101 (Asunercept) blocks both apoptotic and non-apoptotic signaling pathways.

As aforementioned, CD95 can induce a broad range of signaling pathways, with different biological outcomes. This is related to a fine-tuned control of CD95 aggregation, conformation, distribution within plasma membrane sub-domains and post-translational modifications. These parameters rely on the quality of the CD95/CD95L interaction (141). MMPs and ADAMs are responsible for the generation of soluble CD95L, that might promote metastatic occurrence in cancers or stimulate trafficking/activation of immune cells in chronic inflammatory disorders and thus, inhibiting MMP or ADAM activity could represent an attractive therapeutic strategy in these pathologies (**Figure 3**). In addition, inhibition of the non-apoptotic signaling pathways downstream s-CD95L/CD95 interaction might also represent an attractive option to treat certain cancers and chronic inflammatory disorders. Asunercept (initially called APG101) is a decoy receptor encompassing the extracellular region of CD95 fused to the Fc domain of human IgG1. APG101 interacts with CD95L, both transmembrane and soluble forms (**Figure 3**), and abrogates all signals induced by these ligands. Asunercept in phase I/II clinical trials exhibits encouraging therapeutic effect on myelodysplastic syndromes (142) and glioblastoma (143, 144). In addition, the therapeutic value of this drug is also under evaluation (NCT04535674) in COVID-19 patients, in whom CD95L inhibition might protect against the macrophage/neutrophil-driven damage of epithelial cells (145). Although the clinical outcomes of these trials are motivating, it remains that APG101 blocks both apoptotic and non-apoptotic signals, rendering difficult to discriminate the role of each cellular response in the pathogenesis. We recently developed a drug (i.e., peptidomimetic) neutralizing in a selective fashion, the CD95 non-apoptotic pathway (78). This drug, designated DB550, disrupts the CD95/PLCγ1 interaction and the subsequent calcium signaling pathway, which is mandatory for cell migration (77). DB550 injection in SLE-prone mice prevents Th17 cell transmigration in inflamed kidneys and alleviates clinical symptoms (78). These findings support that the selective inhibition of CD95-mediated non apoptotic pathways might turn out sufficient to treat cancers and chronic autoimmune disorders in which s-CD95L is up-regulated (36).

Regarding m-CD95L shedding, another alternative to selectively block the CD95-mediated non-apoptotic signal is to prevent the generation of s-CD95L by inhibiting metalloproteases. Beyond the fact that metalloproteases are pleotropic enzymes, whose inhibition will engender clinical outcomes difficult to predict, an additional concern is the accumulation of membrane-bound CD95L that, might favor the elimination of certain cancer or immune cells, but might also engender undesired tissue damage (**Figure 3**). Finally, another therapeutic approach for cancer patients could be to develop methods to extinguish the CD95 expression itself. Indeed, we recently observed that the elimination of CD95 in triple negative breast cancers induces a pro-inflammatory signal and promote the anti-tumor activity of NK cells (139, 140).

## Author contributions

LD, NG, SB, AC, MJ and PL wrote the original draft. All authors contributed to the article and approved the submitted version.

## Funding

This work was supported by INCa PLBIO (PLBIO 2018-132), ANR PRCE (ANR-17-CE15-0027), and with financial support from ITMO Cancer of Aviesan within the framework of the 2021-2030 Cancer Control Strategy, on funds administered by Inserm.

## Conflict of interest

PL and MJ are involved in patents protecting the use of CD95 or CD95L in chronic inflammatory disorders and cancers WO2014118317; WO2015189236; WO2015158810; WO2015104284; WO2017149012; WO2018130679.

The remaining authors declare that the research was conducted in the absence of any commercial or financial relationships that could be constructed as a potential conflict of interest.

## Publisher’s note

All claims expressed in this article are solely those of the authors and do not necessarily represent those of their affiliated organizations, or those of the publisher, the editors and the reviewers. Any product that may be evaluated in this article, or claim that may be made by its manufacturer, is not guaranteed or endorsed by the publisher.
